# Multilevel Modelling of the Individual and Regional Level Variability in Predictors of Incomplete Antenatal Care Visit among Women of Reproductive Age in Ethiopia: Classical and Bayesian Approaches

**DOI:** 10.3390/ijerph19116600

**Published:** 2022-05-28

**Authors:** Teshita Uke Chikako, Reta Habtamu Bacha, John Elvis Hagan, Abdul-Aziz Seidu, Kenenisa Abdisa Kuse, Bright Opoku Ahinkorah

**Affiliations:** 1 Wondo Genet College of Forestry and Natural Resource, Hawassa University, Hawassa P.O. Box 05, Ethiopia; teshitauke@hu.edu.et; 2 Department of Statistics, Jimma University, Jimma P.O. Box 378, Ethiopia; reta.habtamu@ju.edu.et; 3 Department of Health, Physical Education and Recreation, University of Cape Coast, Cape Coast PMB TF0494, Ghana; 4 Neurocognition and Action-Biomechanics-Research Group, Faculty of Psychology and Sport Sciences, Bielefeld University, Postfach 10 01 31, 33501 Bielefeld, Germany; 5 Centre for Gender and Advocacy, Takoradi Technical University, Takoradi P.O. Box 256, Ghana; abdul-aziz.seidu@stu.ucc.edu.gh; 6 College of Public Health, Medical and Veterinary Sciences, James Cook University, Townsville, QLD 4811, Australia; 7 Department of Statistics, Bule Hora University, Bule Hora P.O. Box 144, Ethiopia; abdisakenenisa40@gmail.com; 8 School of Public Health, Faculty of Health, University of Technology Sydney, Sydney, NSW 2007, Australia; bright.o.ahinkorah@student.uts.edu.au

**Keywords:** antenatal care visit, Bayesian multilevel modeling, EMDHS, reproductive age

## Abstract

Background: Antenatal care is an operational public health intervention to minimize maternal and child morbidity and mortality. However, for varied reasons, many women fail to complete the recommended number of visits. The objective of this study was to assess antenatal care utilization and identify the factors associated with the incomplete antenatal care visit among reproductive age women in Ethiopia. Methods: The 2019 Ethiopian Mini Demographic and Health Survey data were used for this study. Multilevel logistic regression analysis and two level binary logistic regression models were utilized. Results: Around 56.8% of women in Ethiopia did not complete the recommended number of antenatal care visits. Women from rural areas were about 1.622 times more likely to have incomplete antenatal care compared to women from urban areas. Women who had no pregnancy complication signs were about 2.967 times more likely to have incomplete antenatal care compared to women who had pregnancy complication signs. Women who had a slight problem and a big problem with the distance from a health center were about 1.776 and 2.973 times more likely, respectively, to have incomplete antenatal care compared to women whose distance from a health center was not a problem. Furthermore, women who had ever terminated pregnancy were about 10.6% less likely to have incomplete antenatal care compared to women who had never terminated pregnancy. Conclusions: The design and strengthening of existing interventions (e.g., small clinics) should consider identified factors aimed at facilitating antenatal care visits to promote maternal and child health related outcomes. Issues related to urban–rural disparities and noted hotspot areas for incomplete antenatal care visits should be given special attention.

## 1. Introduction

Antenatal care is a pregnancy-related essential health care, which could be given either in a health facility or at home, and is an integral component of maternal and child health [1,2]. It is considered as one of the most important factors for the health of the mother and optimal development of the fetus as well as for preventing or minimizing complications during pregnancy [3]. The complications before the pregnancy period have been the major causes of loss of life and disability among women in the world [3,4]. In 2015, an estimated 303,000 women died as a result of pregnancy- and childbirth-related complications and about 99% of these deaths occurred in low- and middle-income countries, with Sub-Saharan African alone accounting for roughly 66% of these deaths [5].

In addition, maternal mortality is excessively high [6]. In 2017, thousands of women died during and following pregnancy [7]. Ethiopia is among the countries with the highest maternal mortality ratios in sub-Saharan Africa, where the recommended number of Antenatal Care (ANC) visits have still not been achieved in the country [2]. According to the 2016 Ethiopian Demographic and Health survey, pregnancy-related maternal mortality ratios were 412 maternal deaths per 100,000 live births [8]. One of the key strategies in reducing maternal mortality is to access the care given to women during and after pregnancy [9]. Timely and appropriate care given to pregnant women is important for early detection of diseases and provides timely treatment [10,11,12].

The previous World Health Organization’s (WHO) recommended Focused Antenatal Care (FANC) Model. Under normal circumstances, a pregnant woman should have at least four ANC visits [9]. But recently, WHO issued “the 2016 WHO ANC model” with a new series of recommendations to improve the quality of ANC, which in turn help reduce the risk of stillbirths, complications, and ensure a positive pregnancy experience. The new WHO model recommends a minimum of eight contacts [13]. Though the recent 2016 WHO model recommends 8 contacts, the country guideline of Ethiopia still promotes 4 ANCs having slight time differences from the previously WHO recommended FANC model [14]. However, in developing countries, most pregnant women start their first ANC visit later than recommended [12,13]. For instance, globally, only 64% of women receive ANC four or more times throughout their pregnancy. Reports showed slower progress in sub-Saharan Africa than in other regions [15]. Previous research in sub-Saharan Africa showed that different sociodemographic factors, such as age, educational status, wealth index, geographical location (urban-rural), husband’s occupation, marital status, and wealth status, are associated with ANC utilization [16,17,18]. For instance, recent studies in Ethiopia (e.g., [2]), Nigeria (e.g., [19,20]), and other parts of sub-Saharan Africa [21] suggested that the likelihood of not receiving ANC was high among poor women, due to inadequate financing, the non-availability of competent and skilled health care providers in rural areas, distance to ANC facilities, and inadequate or poor-quality health services.

Substantial progress has been made in the past two decades in Ethiopia, where many pregnant women do not receive ANC or receive less than the recommended number of ANC visits and fewer women start ANC at an appropriate time [22]. According to the Ethiopian Demographic and Health Survey (EDHS) 2011 and 2016 reports, 34% and 62%, respectively, of women who had a live birth in the five years before the survey received ANC from a skilled provider at least once for their last birth. In addition, only 19% and 32% of women had four or more ANC visits during their last pregnancy respectively. Furthermore 20% of women made their first ANC visits before the fourth month of pregnancy [8].

The time to first ANC visit, as well as the total number of ANC visit, also affects the quality of the ANC that a pregnant woman receives and not having the recommended ANC service may lead to adverse pregnant outcomes [23]. This is primarily because different services and interventions are available for different gestational ages. ANC visits are vital for the early detection of HIV, malaria, and anemia prophylaxis, and prevention or management of complications [24]. Although scholarly information accounting for incomplete antenatal care services in Ethiopia exists, further research is required to gain a clearer understanding of the issues surrounding incomplete ANC visits in the country. The objective of this study was to assess the prevalence of ANC utilization and identify the factors associated with incomplete ANC among women of reproductive age in Ethiopia.

## 2. Materials and Methods

### 2.1. Source of Data

The data for this study were taken from the Ethiopian Mini Demographic and Health Survey (EMDHS) of 2019. Ethiopia is located in the horn of East Africa. It has nine regions and two self-governing districts (Addis Ababa and Dire Dawa). The survey drew a representative sample of women of reproductive age (15–49), by administering a questionnaire, which was conducted across the country and available from the EDHS database at https://dhsprogram.com/data/available-datasets.cfm (accessed on 1 February 2022). Data collection took place from 21 March 2019, to 28 June 2019 [14]. The first EMDHS were conducted in 2014 and then subsequently in 2019. It was administered at the household level and funded by the USAID in many middle- and low-income countries.

### 2.2. Study Variables

#### 2.2.1. Outcome Variable

The number of antenatal care visits was the outcome variable of this study. The recent WHO report recommends women to have at least eight antenatal care visits during their last pregnancy. However, the Ethiopian mini report shows that data were collected using the old recommendation by the WHO (at least 4 visits) [14]. Following the Ethiopia mini report, incomplete antenatal care can be defined as less than four antenatal care visits during their last pregnancy of a woman aged 15-49. Therefore, antenatal care is captured as dichotomous and coded as 1 if a woman aged 15–49 in Ethiopia had less than four antenatal care visits during their last pregnancy and 0 if otherwise.

#### 2.2.2. Independent Variables

Several predictors, such as residence, women’s education level, family wealth index, pregnancy complication signs, media exposure, ever had a terminated pregnancy, distance from a health center, who decided on the respondent’s heath care, religion, mother’s age, and mother’s occupation were considered in this study as factors associated with incomplete antenatal care.

### 2.3. Statistical Analyses

#### 2.3.1. Multilevel Logistic Regression Model

Since our dependent variable is dichotomous, logistic regression is the popular model to analyze the dataset [13]. This model assumes that all women are independent in the sense that any variables affecting the dependent variable have the same effect in all clusters. Generally, women are nested within the region and lack of independence across levels of nested data as they shared common characteristics. To overcome these problems, multilevel logistic regression model was chosen as the appropriate model. A multilevel logistic regression model can be used to analyze nested sources of variability in hierarchical data, considering of the variability associated with each level of the hierarchy [25]. The study involves a two-level model with different steps for estimation: null and final models (random intercept model). The null model involves no predictors specified at either level. The final model (random intercept model) involves random intercept and explanatory variables from the two levels. In this study, the basic data structure of the two-level logistic regression is a collection of 11 groups (regions), and within group *j* (1, 2,..., 11), a random sample *n_j_* of level-one units (individual women). The outcome variable is given by *Y_ij_*:

$$Y_{ij}={\{\begin{array}{c} 1\;if\;the\;i\mathrm{th}\;women\;in\;j\mathrm{th}\;region\;have\;incomplete\;ANC\;\\ 0\;if\;the\;i\mathrm{th}\;women\;in\;j\mathrm{th}\;region\;have\;complete\;ANC \end{array}}_{\;}$$

*P_ij_* = *P*(*Y_ij_* = 1|*X_ij_*, *U_ij_*) is the probability of having incomplete *ANC* for the *i*th women in the *j*th region and 1 − *P_ij_* = *P*(*Y_ij_* = 0|*X_ij_*), is the probability of having complete *ANC* for the *i*th women in the *j*th region, where *X_ij_* stands for set of fixed independent variable and *U_ij_* stands for the random part of the model.

#### 2.3.2. The Null Model

This model is given as:

$$Y_{ij}=\beta_{0}+U_{0j}+\varepsilon_{0ij}\;,\;\varepsilon_{0ij}\sim N\left(0,\;\sigma_{\varepsilon }^{2}\right)\;,\;U_{0j}\sim N{\left(0,\;\sigma_{u}^{2}\right)}_{\;}$$

where the index *i* indicates individual women, *j* indicates region, *U*_0*j*_ is the level two error, $\varepsilon_{0ij}$ is the level one error, *β*_0_ is interpreted as the overall average of antennal care visit status, and *Y_ij_* is antennal care visit status of *i*th women in *j*th region.

This model decomposes the variance at two levels to assess how much of the variation is due to individual themselves and how much is due to region. The interclass correlation (ICC) is given as:

$$\mathrm{ICC}\;=\rho =\frac{\sigma_{u}^{2}}{\sigma_{\varepsilon }^{2}+\sigma_{u}^{2}}=\frac{\sigma_{u}^{2}}{\sigma_{Y}^{2}}$$

This measures the correlation between variables on the same level or proportion of group level variance compared to total variance. It can also be interpreted as the expected correlation between two randomly chosen units in the same level and to check within and between variations of regions of women’s antennal care visit status. Next, predicators of the two levels are added to the null model to obtain the final model known as the random intercept model and the fixed explanatory model [26]. The random intercept model expresses the log odds, i.e., the logit (*P_ij_*), as a sum of linear functions of the explanatory variables.

#### 2.3.3. The Final Model

The final multilevel model can be written as:

$$Y_{ij}=\beta_{0}+\beta_{1}X_{1ij}+\ldots +\beta_{m}X_{mij}+\varepsilon_{ij}+U_{j}\;$$

where *Y_ij_* is women’s antenatal care status, $\varepsilon_{ij}$ is the level one variance, *U_j_* is the level two variance, and *m* is the household-level explanatory variables. Since the multilevel logistic regression is nonlinear, the above model can be given by logit transformation of its probability (*logit* (*P_ij_*)) as follows:

$$logit\left(P_{ij}\right)=log\left[\frac{P_{ij}}{1-P_{ij}}\right]=\beta_{0}+\beta_{1}X_{1ij}+\ldots +\beta_{m}X_{mij}+U_{j}\;$$

Parameter estimation in multilevel logistic regression is not direct like an ordinary regression model. To estimate unknown parameters, we applied marginal maximum likelihood [27]. This method of parametric estimation is most popular in the multilevel logistic regression model.

### 2.4. Bayesian Multilevel Logistic Regression Model

The three pillars Bayesian analysis were the likelihood function, which reflect information about the parameters contained in the data, the prior distribution, which quantifies what is known about the parameters before observing data, and the prior distribution and likelihood can be easily combined to form the posterior distribution, which represents the total knowledge about the parameters after the data have been observed [28,29,30]. The study considers normal for fixed effect and inverse gamma for random effect as prior distribution. The likelihood contribution from the *i*th subject in the *j*th group is Bernoulli and given by:

$$Bernoulli\;\left(P_{ij}\right)=P_{ij}^{y_{ij}}{\left(1-P_{ij}\right)}^{1-y_{ij}}\;$$

where *P_ij_* represents the probability of the event for subject *i* in the *j* group, which has covariate vector *x_ij_*, and *y_ij_* indicates the presence, *y_ij_* = 1, or absence, *y_ij_ =* 0, of the event for that subject. Thus, the likelihood contribution for the *i*th subject in the *j*th region is:

$$L\left(y|\beta_{i},\sigma_{u}^{2}\right)={\left(\frac{e^{\beta_{0}+\beta_{1}X_{1ij}+\ldots +\beta_{m}X_{mij}+U_{j}}}{1+e^{\beta_{0}+\beta_{1}X_{1ij}+\ldots +\beta_{m}X_{mij}+U_{j}}}\right)}^{y_{ij}}{\left(1-\frac{e^{\beta_{0}+\beta_{1}X_{1ij}+\ldots +\beta_{m}X_{mij}+U_{j}}}{1+e^{\beta_{0}+\beta_{1}X_{1ij}+\ldots +\beta_{m}X_{mij}+U_{j}}}\right)}^{1-y_{ij}}$$

Since individual subjects in the group are assumed independent from each other, the likelihood function over a dataset of n subjects in the 11 region is then:

$$L\left(y|\beta_{i},\sigma_{u}^{2}\right)=\overset{n}{\underset{i=1}{\prod }}\overset{11}{\underset{j=1}{\prod }}\left[{\left(\frac{e^{\beta_{0}+\beta_{1}X_{1ij}+\ldots +\beta_{m}X_{mij}+U_{j}}}{1+e^{\beta_{0}+\beta_{1}X_{1ij}+\ldots +\beta_{m}X_{mij}+U_{j}}}\right)}^{y_{ij}}{\left(1-\frac{e^{\beta_{0}+\beta_{1}X_{1ij}+\ldots +\beta_{m}X_{mij}+U_{j}}}{1+e^{\beta_{0}+\beta_{1}X_{1ij}+\ldots +\beta_{m}X_{mij}+U_{j}}}\right)}^{1-y_{ij}}\right]$$

A non-informative prior distribution that is used to express complete ignorance of the value before the data are collected was considered. We prefer non-informative because no value is favored over any other, and they are also described as diffuse or at prior due to their reason and their shape. The log odd of fixed effect is approximately normal and the random effect is random. This leads the authors to use normal distribution prior for the fixed effect $(\beta_{i}$) and Inverse gamma distribution prior for random effect ($\sigma_{i}^{2}$).

Combining the likelihood function with the prior distribution on ($\beta ,\sigma_{u}^{2}$) and the full conditional distribution for unknown parameters, the posterior distribution can be written as:

$$f\left(\beta ,\sigma_{u}^{2}|y\right)=L\left(y|\beta ,\sigma_{u}^{2}\right)\times \pi \left(\beta \right)\times \pi \left(\sigma_{u}^{2}\right)$$

After posterior distribution is determined, the assessment of algorithm convergence was checked to identify whether chain has reached its stationary or not. The term convergence of an algorithm refers to whether the algorithm has reached its equilibrium (target) distribution. If the algorithm has reached its equilibrium, then the generated sample comes from the right target distribution. Thus, monitoring the convergence of the algorithm is crucial for generating effective results from the posterior distribution. Therefore, to monitor the convergence of the algorithm, we used the most popular and straight forward convergence assessment methods such as Rhat, Bulk effective sample size (Bulk_ESS), and Tail effective sample size (Tail_ESS). If Rhat = 1 and Bulk_ESS and Tail_ESS were greater than 1000, then we have evidence that the chain has converged.

### 2.5. Software

The data analysis was done using R statistical software ((Version 4.0.4, Foundation for Statistical Computing, Vienna, Austria). The authors used R software with lme4 package for the classical multilevel logistics and R software with brms package for the Bayesian multilevel logistics analysis. Based on previous literature [30], we used non informative normal prior distribution with mean = 0 and precision = 0.001 for the fixed effect and inverse gamma distribution (0.1, 0.1) for the random effect, with iteration = 20,000, warm-up = 2000 (number of iterations that was discarded), chains = 2, initials (the starting values of the iterations) = 0, cores (specifies the number of cores used for the algorithm) = 2, and adapt delta (controls divergent transition) = 0.95. Summary statistics were carried out from the posterior distribution after the model was converged and the 95% credible interval was used for the test of significance.

### 2.6. Ethics Approval

Ethical clearance was obtained by the Institutional Review Board of ICF International. Permission was also sought from each woman during the fieldwork. The authors requested and officially received approval from the DHS Program to use the dataset for this study. Detailed information about the DHS data usage and ethical standards are available at http://goo.gl/ny8T6X (accessed on 1 February 2022).

## 3. Results

### 3.1. Descriptive Results 

The total number of women aged 15 to 49 years covered in this study was 3190. From these, 1812 women aged 15–49 years (56.8%) were not following the recommended number of antenatal care visits (Table 1).

### 3.2. Result of the Variance Component Model

In this model, we first fitted a simple model with no predictors, (i.e., an intercept-only model) that predicted the probability of incomplete antenatal care status. This model contains no explanatory variables, and it can be considered as a parametric version of assessing heterogeneity among regions with respect to incomplete ANC visits (Table 2).

From Table 2, we consider the variance of the random factor (between region variance) as significantly different from zero, which indicates that there are regional differences in incomplete ANC.

To get an idea of how much of variation in incomplete antenatal care was attributable to the individual level and regional level, it is useful to see the intra-region correlation coefficient (ICC). ICC = 0.081 indicates that around 8.1% of variation in incomplete ANC among women is due to the variation across (between) regions, whereas the remaining 91.9% was attributable to individual level (i.e., within-region) differences.

### 3.3. Result of the Classical Multilevel Model

Table 3 results show that residence, women’s education level, family wealth index, pregnancy complication signs, media exposure, ever had a terminated pregnancy, distance from a health center, and who decided on the respondent’s heath care are significant predictors of incomplete ANC among women aged 15–49 years in Ethiopia, while religion, mother’s age, and mother’s occupation were non-significant predictors of incomplete ANC among women aged 15–49 years in Ethiopia.

Women from rural areas were about 1.622 times more likely to have incomplete ANC visits compared to women from urban areas. Additionally, women who ever had a terminated pregnancy were about 10.6% less likely to have incomplete ANC compared to those women who never had a terminated pregnancy. The results also indicate that women who had secondary and higher education were about 20.5% and 31.1% less likely to have incomplete ANC compared to those women who do not have formal education. Table 3 also shows that women who had slight problem and big problem with distance from a health center were about 1.776 and 2.973 times more likely, respectively, to have incomplete ANC compared to women who saw distance to a health center as not a problem.

Table 3 also revealed that rich women were 15.4% less likely to have incomplete ANC compared to poor women. Additionally, women who had no pregnancy complication signs were about 2.967 times more likely to be incomplete ANC compared to the women who did have pregnancy complication signs. Likewise, women who were exposed to all media sources at least once a week were about 28.2% and 8.7% less likely to have incomplete ANC compared to women who have no media exposure at all. Concerning a person who usually decides on the respondent’s health care, respondents whose health care was decided by the respondent and husband and respondent and another person were 29.7% and 42.4% less likely to have incomplete ANC compared to respondents whose health care was decided by the respondent alone.

From the result of the variance component model and random intercept model, AIC = 13,149.9 and BIC = 13,411.7 for the random intercept model were the smallest compared to the variance component model. This estimation shows that the random intercept model better fits the data to predict the factors for incomplete ANC among women aged 15–49 in Ethiopia.

The contrast between the two prominent statistical views, classical and Bayesian approaches, should be based on the better fit model (random intercept model), and then, a rigorous decision should be made for having accurate implementation.

### 3.4. Results of the Bayesian Multilevel Model

From Table 4, the Rhat value is 1, and all effective sample sizes (both Bulk_ESS and Tail_ESS) are greater than 1000, which suggests that the model was converged and reached its equilibrium. Table 4 revealed that residence, women’s education level, family wealth index, pregnancy complication signs, media exposure, ever had a terminated pregnancy, distance from a health center, and who decided on the respondent’s heath care were significant predictors of incomplete ANC of women aged 15–49 years in Ethiopia (since their 95% confidence intervals does not contain zero). Religion, mother’s age, and mother’s occupation were not significant predictors of incomplete ANC of women aged 15–49 years in Ethiopia (since their 95% confidence intervals contains zero).

The results of the classical multilevel and Bayesian multilevel show that the data were well fitted and the results of both approaches were almost consistent, but almost all parameters in the Bayesian result had small standard errors compared to the corresponding classical multilevel logistic model. This is because the Bayesian analysis gives additional solutions as the posterior distribution of the parameters.

## 4. Discussion

This study investigates the predictors of incomplete ANC among women aged 15–49 years in Ethiopia. The results revealed that residence, women’s education level, family wealth index, pregnancy complication signs, media exposure, ever had a terminated pregnancy, distance from a health center, and who decided on the respondent’s heath care were found to be significant predictors of incomplete ANC of women aged 15–49 years in Ethiopia, while religion, mother’s age, and mother’s occupation were non-significant predictors of incomplete antenatal care of women aged 15–49 years in the country.

The study shows residence has significant relation with incomplete ANC and the utilization of ANC varies with place of residence, such that women from rural areas have less ANC visits than women from urban areas. This finding is consistent with other studies done in Ethiopia and Nigeria [2,17,31]. The variations noted suggest that urban women could have more access to health services and have considerable information and education about ANC than their rural counterparts. This finding suggests that health service inequity is preventable and could be biased by the systematic dissimilarities in the health of residents rooted in social determinants of health [32]. Therefore, health equity and social determinants become crucial components of the postmillennium goal and sustainable development global agendas as a part of progressive accomplishment for collective health coverage [33,34]. The social determinants of health, such as the conditions in which people are born, grow, work, and live, including the wider set of social systems, such as economic policies and systems, development agendas, social norms, social policies, and political systems, shape the conditions of peoples’ daily life and bring health inequity [35]. Worldwide, the inequalities in the distribution of health facilities are reflected by the higher proportion of ANC use in urban centers as compared to rural areas [33,34,36]. These discrepancies might be because of the local inaccessibility of obstetric care services in rural areas as well as disparities in some district administrations. Therefore, stakeholders and other service providers must work together for improving societies’ easy access to healthcare services.

Similarly, women who had ever terminated pregnancy had more ANC visits than those who had never terminated pregnancy. This finding was supported by a study carried out in Ethiopia, Malawi, and other low-income and middle-income countries [37,38,39]. Usually, illness experienced, complications associated with past pregnancy termination, and perceived susceptibility to illnesses connected to pregnancy termination could trigger more antenatal care utilization of women in reproductive age [40,41,42].

Women with a secondary/higher level of educational and high wealth index were less likely to have incomplete antenatal care compared to women with no formal education. Women with a higher educational level are more likely to be knowledgeable about the benefits of ANC services. Logically, higher-educated women could be more flexible, open to new ideas, and make appropriate decisions about their families’ health, including ANC [43]. Furthermore, when women are of low socio-economic standing (i.e., education and wealth) and do not have adequate resources or limited resources could create inadequate resource allocation to women and to ANC; hence, their adherence to ANC could be minimized [44,45,46]. Therefore, high educational level and wealth could serve as a protective mechanism against incomplete antenatal care [47,48,49]. The study discovers that having wanted pregnancy was significantly associated with the odds of having fewer antenatal care visits as compared to unwanted pregnancies. This result was similar with other studies done in Tanzania, Nigeria, and Ghana, as women who had a wanted pregnancy were more likely to have antenatal care [50,51,52]. Women with wanted pregnancies might feel a higher desire for a healthy pregnancy and childbirth, and thus, may pay greater attention to antenatal care services.

Similar to previous studies [53,54,55], the distance to the nearest health center with ANC-providing facilities in a typical region was significantly associated with the odds of having incomplete antenatal care. For example, traveling long distances and having good access routes to ANC centers could restrict regular attendance for ANC services. Conversely, those women who live close to obstetric health facility were more likely to use antenatal care services [56]. This finding indicates that geographic accessibility, measured in either distance or travel time, has a greater influence on maternal health service utilization. Furthermore, media exposure might also play a vital role in increasing the use of more antenatal care and other maternal health services. In this study, women who had almost all and at least once a week access to mass media were more likely to have complete antenatal care, which is recommended by WHO, than those who had no media exposure at all. This result was in line with previous studies done in Nepal, Ghana, and Ethiopia [57,58,59]. Utilizing mass media to spread an antenatal care message could be an effective approach to improve the uptake of early initiation of the recommended antenatal care. The frequency of mass media (e.g., regular listening of radio messages) has been shown to positively impact on the utilization of maternal healthcare services, including the regularity of ANC visits by women [57].

### Strength and Limitations of the Study

The strength of this study was the incorporation of a large dataset that was nationally-representative. This could increase the generalization of the study finding to the whole region of Ethiopia. However, the cross-sectional design used restricted causal interpretation of the findings, but only associations. The study was based on a secondary dataset, which limits control over the types of variables available. Hence, the data analyses used were restricted to only the variables captured in the DHS. It is, therefore, possible that other interconnected variables were overlooked during the survey. For example, future research on ANC should use the recent WHO recommendations of 8 visits or contacts as a standard reference for comparison. Due to the self-reported nature of the survey, issues related to recall and social desirability biases cannot be ignored in this study.

## 5. Conclusions

The results show that the prevalence of incomplete ANC among women aged 15–49 years was 56.8% in Ethiopia. The major determinants associated with incomplete ANC in Ethiopia were residence, ever had a terminated pregnancy, women’s education level, distance from a health center, wealth index, pregnancy complication signs, media exposure, and who decided on the respondent’s health care. To enhance the number of ANC visits, the design and strengthening of existing interventions (e.g., small clinics) should consider the identified factors when facilitating antenatal care visits to promote maternal and child health-related outcomes. Issues related to urban–rural disparities and noted hotspot areas for incomplete ANC visits should be given special attention.

## Figures and Tables

**Table 1:** Descriptive result of outcome variables.

Variable	Less than 4 Visit		Greater or Equal to 4 Visits	
	Count	Percent	Count	Percent
Number of antenatal care visits	1812	56.8%	1378	43.2%

**Table 2:** Result of the variance component model.

Fixed Effect	Estimate	Std. Error	Z-Value	*p*-Value
Intercept	−0.4562	0.1640	−2.782	0.028
Random Effect				
${\hat{\sigma }}_{u}^{2}$	0.29	0.065	4.462	0.003
ICC (*ρ*)	0.081			

AIC = 13,419.7, BIC = 13,434.3, logLik = −6707.9, deviance= 13,415.7.

**Table 3:** Result of the classical multilevel model.

Variable	Category	Estimate	Std. Error	Z-Value	*p*-Value	Odds Ratio
Intercept		0.591	0.120	4.92	0.0026	
Residence	Urban (ref.)					
	Rural	0.497	0.089	5.545	0.0002	1.622
Ever had a terminated pregnancy	No (ref.)					
	Yes	−0.166	0.076	−2.184	0.0487	0.894
Women’s education level	No Formal Education (ref.)					
	Primary	−0.056	0.058	−0.964	0.334	0.658
	Secondary	−0.209	0.101	−2.076	0.038	0.795
	Higher	−0.424	0.144	−2.932	0.003	0.689
Religion	Orthodox (ref.)					
	Muslim	0.104	0.264	0.395	0.692	1.724
	Protestant	0.009	0.088	0.096	0.923	1.810
	Other	0.082	0.073	1.119	0.263	1.737
Distance from a health center	No problem (ref.)					
	Slight problem	0.566	0.223	2.538	0.025	1.776
	Big problem	0.108	0.048	2.251	0.038	2.973
Wealth index	Poor (ref.)					
	Middle	0.088	0.056	1.583	0.113	0.771
	Rich	−0.267	0.094	−2.831	0.014	0.846
Pregnancy complication signs	Yes (ref.)					
	No	0.132	0.05	2.641	0.002	2.967
Media exposure	Not at all (ref.)					
	Less than Once a week	−0.042	0.057	−0.725	0.468	0.667
	At least Once a week	−0.244	0.056	−4.348	0.0001	0.718
	Almost all	−0.152	0.071	−2.145	0.032	0.913
Mother’s age	15–19 (ref.)					
	20–24	−0.103	0.115	−0.898	0.369	0.855
	25–29	−0.052	0.118	−0.437	0.662	0.862
	30–34	−0.101	0.122	−0.898	0.369	0.772
	35–39	−0.155	0.142	−1.094	0.274	0.812
	40–44	0.153	0.188	0.816	0.415	0.767
	45–49	−0.311	0.175	3.173	0.075	0.732
Who decided on the respondent’s health care	Respondent alone (ref.)					
	Respondent and husband	−0.131	0.057	−2.29	0.037	0.703
	Respondent and another person	−0.186	0.070	−2.635	0.008	0.576
	Husband alone	0.073	0.056	1.687	0.057	1.194
Occupation	Not working (ref.)					
	Managerial	0.055	0.151	0.366	0.714	1.340
	Clerical	0.06	0.067	0.883	0.377	1.167
	Sales	−0.174	0.317	−0.550	0.582	1.419
	Agricultural	−0.292	0.171	−1.708	0.088	1.424
	Other	0.108	0.353	0.094	0.760	1.114
${\hat{\sigma }}_{u}^{2}$		0.271	0.068			
ICC (*ρ*)		0.076				

AIC = 13,149.9, BIC = 13,411.7, logLik = −6538.9, deviance = 13,077.9.

**Table 4:** Result of the Bayesian multilevel model.

Variable	Category	Estimate	Std. Error	95% CI		Rhat	Bulk_ESS	Tail_ESS
				Lower	Upper			
Intercept		0.594	0.118	0.341	0.847	1.00	8795	8954
Residence	Urban (ref.)							
	Rural	0.509	0.086	0.368	0.651	1.00	11,542	10,598
Ever had a terminated pregnancy	No (ref.)							
	Yes	−0.154	0.072	−0.306	−0.051	1.00	15,874	12,698
Women’s education level	No Formal Education (ref.)							
	Primary	−0.047	0.055	−0.168	0.174	1.00	18,596	17,849
	Secondary	−0.203	0.091	−0.328	−0.078	1.00	19,872	19,877
	Higher	−0.422	0.141	−0.563	−0.181	1.00	20,187	19,874
Religion	Orthodox							
	Muslim	0.103	0.265	−0.105	0.218	1.00	5847	4526
	Protestant	0.010	0.09	−0.154	0.169	1.00	3485	4596
	Other	0.085	0.071	−0.251	0.336	1.00	6789	6875
Distance from a health center	No problem (ref.)							
	Slight problem	0.568	0.221	0.316	0.818	1.00	10,564	9865
	Big problem	0.104	0.045	0.012	0.198	1.00	11,895	9987
Wealth index	Poor (ref.)							
	Middle	0.089	0.051	−0.125	0.053	1.00	15,784	12,589
	Rich	−0.263	0.092	−0.378	−0.148	1.00	13,589	12,485
Pregnancy complication signs	Yes (ref.)							
	No	0.136	0.048	0.051	0.221	1.00	21,448	19,874
Media exposure	Not at all (ref.)							
	Less than Once a week	−0.045	0.053	−0.179	0.089	1.00	12,548	12,478
	At least Once a week	−0.241	0.054	−0.346	−0.136	1.00	12,457	12,478
	Almost all	−0.150	0.069	−0.195	−0.103	1.00	13,487	13,154
Mother’s age	15–19 (ref.)							
	20–24	−0.101	0.112	−0.145	0.139	1.00	9534	9845
	25–29	−0.054	0.116	−0.168	0.122	1.00	7846	7256
	30–34	−0.103	0.121	−0.196	0.128	1.00	5487	6547
	35–39	−0.152	0.141	−0.235	0.112	1.00	4514	3984
	40–44	0.150	0.185	−0.224	0.145	1.00	6589	6847
	45–49	−0.313	0.176	−0.487	0.189	1.00	5478	5894
Who decided on the respondent’s health care	Respondent alone (ref.)							
	Respondent and husband	−0.129	0.055	−0.194	−0.064	1.00	12,457	12,254
	Respondent and another person	−0.182	0.069	−0.281	−0.084	1.00	11,486	11,345
	Husband alone	0.071	.0540	−0.045	0.148	1.00	10,648	10,245
Occupation	Not working (ref.)							
	Managerial	0.052	0.149	−0.115	0.159	1.00	9845	9632
	Clerical	0.061	0.066	−0.068	0.186	1.00	5478	5148
	Sales	−0.173	0.316	−0.246	0.048	1.00	6487	6245
	Agricultural	−0.293	0.168	−0.375	0.082	1.00	3487	3987
	Other	0.105	0.351	−0.154	0.345	1.00	4578	5986
${\hat{\sigma }}_{u}^{2}$		0.283	0.064			1.00	2584	3025
ICC (*ρ*)		0.079						

## Data Availability

The dataset used for the study is available at: https://dhsprogram.com/data/available-datasets.cfm (accessed on 1 February 2022).
